# Xiaoyaosan modulates gut-brain metabolic pathways and brain microstructure in depression: a multi-omics insight

**DOI:** 10.1186/s13020-025-01212-z

**Published:** 2025-10-01

**Authors:** Wen-zhi Hao, Yan-ru Sun, Ying-ren Zhang, Rong-yan-qi Wang, Wen Ning, Lu Wang, Dong-dong Liu, Yong-xin Li, Jun-qing Huang, Jia-xu Chen

**Affiliations:** 1 Guangzhou Key Laboratory of Formula-Pattern of Traditional Chinese Medicine, School of Traditional Chinese Medicine, Jinan University, Guangzhou, China; 2 School of Traditional Chinese Medicine, Beijing University of Chinese Medicine, Beijing, China; 3 Nutrition and Bromatology Group, Department of Analytical and Food Chemistry, Faculty of Sciences, Universidade de Vigo, Ourense, Spain

**Keywords:** Xiaoyaosa, Depression, Gut-brain axis, Metabolic reprogramming, Chronic restraint stress

## Abstract

**Background:**

Depression is closely associated with metabolic disorders in the gut-brain axis. Our previous studies using antibiotics (ABX)-treated mice and germ-free mice models demonstrated that Xiaoyaosan (XYS) alleviates depression by modulating metabolic pathways involved in gut-brain interactions. However, the key metabolic pathways remain to be fully characterized.

**Study design:**

We enriched relevant metabolic pathways and analyzed the correlation between depressive-like behaviors and these pathways. We investigated the effects of XYS on metabolic pathways associated with chronic restraint stress (CRS)-induced depression and innovatively incorporated spatial dimension analysis. We further investigated the impact of these metabolic differences on brain microstructure in depression and the recovery situation after the intervention with XYS.

**Methods:**

Spatial metabolomics and multi-omics integration have been applied to explain the mechanisms behind behavioral changes. To comprehensively assess the role of XYS in gut-brain metabolic reprogramming, we innovatively employed an integrated multi-omics approach, including the 16S rRNA sequencing, metabolomic analyses, AFADESI-MSI analysis, and brain diffusion tensor properties analysis.

**Results:**

We observed that XYS could decrease the relative abundances of *Desulfovibrio*, *Erysipelatoclostridium*, *Parasutterella* and significantly increase the relative abundances of *Dubosiella*, *Akkermansia*, and regulate the glycerophospholipid metabolism and tryptophan metabolism. Spatial and quantitative differences in lipid metabolism, tryptophan metabolism, glutamate/glutamine metabolism, acetylcholine and adenosine metabolism in the brain were observed after XYS treatment. Diffusion tensor analysis further demonstrated that treatment with XYS effectively suppressed the loss of neural integrity in the medial prefrontal cortex and hippocampus caused by chronic restraint stress.

**Conclusion:**

These findings suggest that the antidepressant efficacy of XYS may involve the regulation of gut microbiota and microbial metabolites, improve synaptic loss, influencing the spatial distribution and concentration of brain-specific functional metabolites and reprogramming gut-brain axis metabolism. The application of spatial metabolomics and multi-omics integration can provide new ideas for the research of traditional Chinese medicine.

**Supplementary Information:**

The online version contains supplementary material available at 10.1186/s13020-025-01212-z.

## Introduction

Mental disorders pose a significant challenge to global public health, with depression being the most prevalent and representative clinical mental illness [1]. The pathogenesis of depression is closely intertwined with systemic metabolic disorders, encompassing metabolic disorders in the central nervous system, gastrointestinal tract, and peripheral blood observed in individuals [2–4]. The chemical messengers involved in the metabolic process, including serotonin, norepinephrine, dopamine and other neurotransmitters and metabolic chemicals such as lipids and amino acids, are responsible for maintaining the homeostasis of brain function and facilitating communication among brain cells. Modulation of these neurotransmitters serves as a fundamental mechanism underlying numerous current antidepressant treatments [5–7]. Intriguingly, the biosynthesis and metabolic transport of these metabolites, which are pivotal in brain function, exhibit a close correlation with gut microbiota and microbial enzymes. It is unequivocally evident that the maintenance of gut-brain metabolic homeostasis serves as a crucial determinant in preserving the overall homeostasis of the central nervous system [8, 9]. However, most research on the alleviation of depression through medication or natural products often focuses on the metabolism of the entire brain alone or metabolism locally in the gut and blood, neglecting investigations pertinent to gut microbiota and the integrated gut-brain metabolic reprogramming. Currently, understanding of the metabolic alterations in the gut-brain axis that are associated with depression remains limited. In particular, the relationship between modifications in gut microbiota and microbial metabolites, and the expression patterns as well as tissue-specific distribution of functional metabolites within brain tissue, necessitates further elucidation. Consequently, unraveling the metabolic reprogramming of the gut-brain axis in the context of depression holds significant importance.

In recent years, it has become widely accepted that depression was characterized by abnormal function and structure in brain areas such as medial prefrontal cortex (mPFC), and hippocampus [10, 11]. Meanwhile, depression harbored a distinct gut microbial composition that significantly differed from the normal control [12]. Contemporary neuroimaging research on the brain-gut axis predominantly focuses on elucidating the intricate associations between gastrointestinal metabolic processes and cerebral functional connectivity/neuroanatomical architecture [13–15]. The microbiome-driven behavioral changes are accompanied by corresponding changes in neural tissue microstructure [15]. From these previous studies, we can see that neuroimaging techniques can enable researchers to investigate brain function and structure in response to alterations in the gut microbiota. A picture of the physiological underpinnings of these gut-brain associations can be provided. Recent studies also showed that abnormalities in neurotransmitters are implicated in the pathogenesis of psychiatric disorders [16]. There are some relationships between the brain neuroimaging changes and the altered neurotransmitters in depression [17, 18]. The synthesis and degradation processes of neurotransmitters produce various metabolites. The release and reuptake of neurotransmitters require a large amount of energy, which leads to changes in energy metabolites in brain tissue. There are complex interactions between brain neurotransmitters and metabolites of brain tissue, which jointly maintain the normal operation of brain functions. And also, network analysis showed correlations between the abundance of several microbiome and metabolites in the brain [19, 20]. Thus, research on the brain neuroimaging changes corresponding to the brain regions with abnormal metabolites in depression can deepen our understanding of the metabolic reprogramming of the gut-brain axis.

Epidemiological evidence supports the positive impact of phytochemical intake from herbal medicine, either in pharmaceutical or nutraceutical form, on the risk of depression [21]. Xiaoyaosan (XYS) has an extensive history of medicinal use in Asian such as China, Japan, and South Korea, as well as in European countries like the United Kingdom, the Netherlands, and Germany, spanning over three decades [22, 23]. The formula composition for XYS includes eight herbs, *Bupleurum chinense DC.*, *Angelica sinensis (Oliv.) Diels*, *Paeonia lactiflora Pall.*, *Atractylodes macrocephala Koidz.*, *Wolfiporia extensa (Peck) Ginns*, *Glycyrrhiza uralensis Fisch.*, *Mentha canadensis*, and *Zingiber officinale* in a ratio of 5:5:5:5:5:4:1:5 [24]. XYS has been documented in the European Pharmacopoeia and partially in the British Pharmacopoeia [25], its efficacy in mitigating depression-like behaviors is well-established. The mechanisms driving these effects include an increase in hippocampal serotonin (5-HT) concentration, decreased glutamate levels in the hippocampus, correction of the intestinal microbiome disorders and improvements in metabolic abnormalities [26–28]. However, existing studies primarily concentrate on the impact of XYS on specific functional metabolites and their downstream mechanisms. The all-encompassing role of XYS in regulating gut-brain metabolic reprogramming, particularly in the comprehensive regulation of intestinal flora, intestinal metabolites, and brain functional metabolites, remains to be fully elucidated. This informational void impedes a comprehensive grasp of the alterations in gut-brain metabolic changes metabolism throughout the development of depression and the potential overarching regulatory mechanism of XYS.

Constructing a metabolic profile of functional metabolites with spatial distribution in biosamples is essential for understanding the tissue-specific molecular mechanisms underlying depression. In this investigation, we assessed the impact of XYS on the behavior of depressed mice and employed gut microbiome, metabolomics, mass spectrometry imaging spatial metabolomics technology and diffusion tensor imaging (DTI) technology to identify gut microbiota, microbial metabolites, differential brain tissue-specific metabolites, brain tissue diffusion properties and metabolic pathways associated with chronic restraint stress (CRS)-induced depression and XYS treatment. Furthermore, we enriched the metabolic pathways and examined the correlation between depressive-like behaviors and these pathways. Notably, we pioneered the exploration of XYS regulation on gut flora, microbial metabolites and brain tissue-specific functional metabolites as a whole using gut microbiome, metabolomics combined mass spectrometry imaging. Furthermore, we analyzed the correlation between changes in gut microbes, microbial metabolites and the distribution and expression of functional metabolites in the brain, providing insights into the antidepressant pharmacodynamic mechanism of XYS based on spatial metabolomics technology. This study enabled us to visually illustrate the gut-brain metabolic alterations following XYS treatment, offering valuable insights into the onset and progression of depression and contributing to guiding its therapeutic interventions.

## Materials and methods

### Animals

Male SPF C57BL/6 mice, aged 8 weeks and weighing 18–22 g, were procured from the Experimental Animal Center of Guangdong Province (SYXK (Yue) 2017–0174). The mice were maintained in controlled conditions, with a temperature of 21 ± 2 °C, humidity ranging between 30 and 40%, and a 12-h light–dark cycle. All procedures involving animals were conducted in accordance with the guidelines established by the Institutional Animal Ethics Committee (Approval ID: 20230413–02). Body weight were systematically recorded every 4 days.

### Chronic restraint stress (CRS) induced depression

**Fig. 1:**
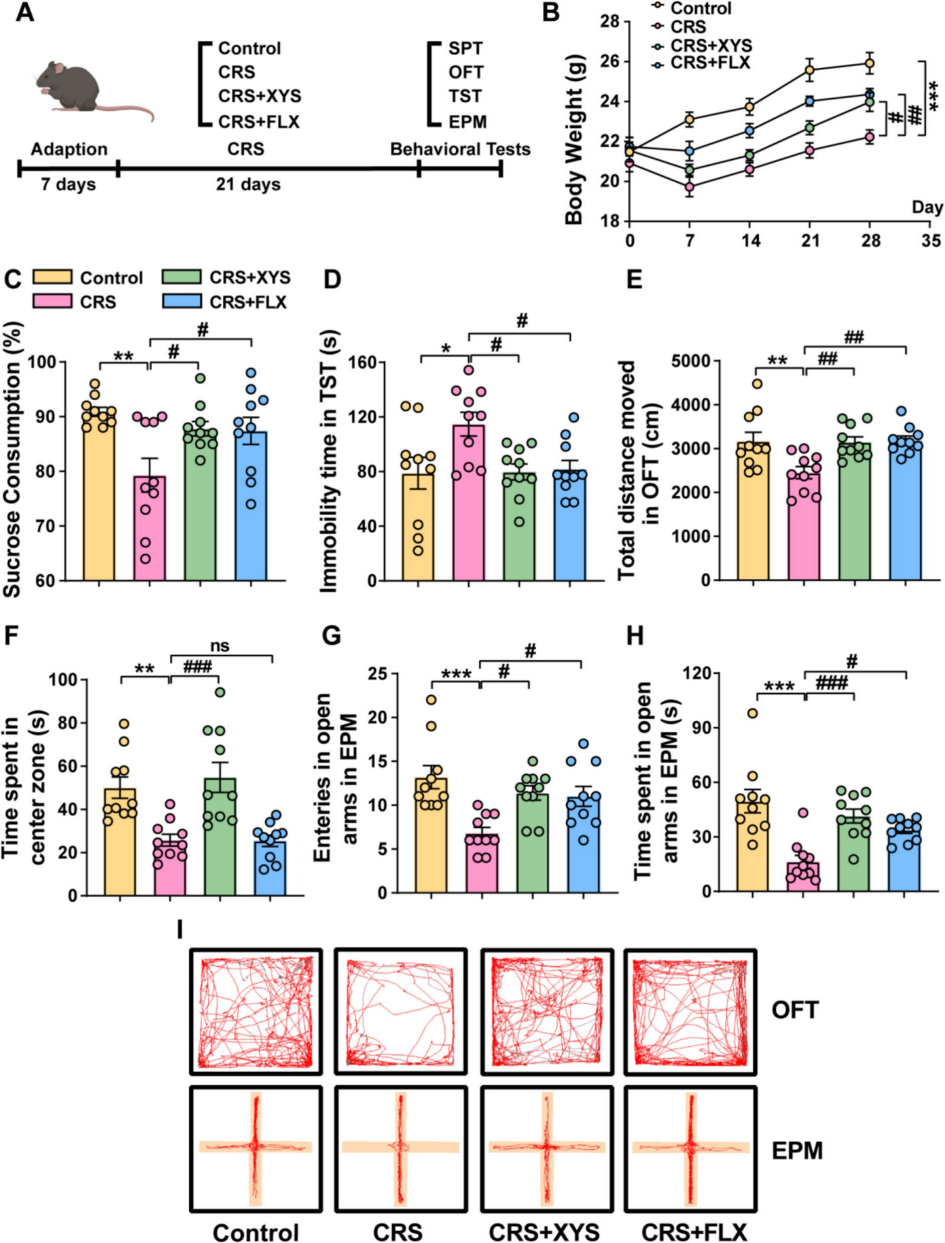
XYS significantly increased the gain of body weight and regulated the abnormal behaviors of depressed mice. **A** The experimental protocol employed in this study. **B** The change of body weight. **C** The sucrose preference rate in SPT. **D** The immobility time in TST. **E** The total distance moved in OFT. **F** The time spent in center zone in OFT. **G** The entries into the open arms in EPM. **H** The time spent in open arms in EPM. **I** The motion trajectory data of mice in OFT and EPM. Data represent the mean ± SEM of ten mice in each group. **P < 0.01 and ***P < 0.001 versus the control group; ^#^*P* < 0.05 and ^##^*P* < 0.01 versus the CRS group

After a 7-day adaptation period, mice were allocated into four groups (N = 15): Control group (Control), Chronic restraint stress group (CRS), XYS treatment group (CRS + XYS), and Fluoxetine treatment group (CRS + FLX). To induce the depression model, mice in the CRS group were confined in plastic tubes with limited airflow for 6 h daily over a 21-day period [29]. The restraint apparatus was composed of transparent plastic tubes with a size of 11 cm × 3 cm. The plastic tube consists of five vent holes, including four at the front end and one at the rear end, to ensure the normal breathing of the mouse. In the control and CRS groups, mice received a daily gavage administration of 0.9% saline at 0.1 ml/10 g body weight. The XYS and Fluoxetine groups were administered a daily dose of 0.658 g/kg/d XYS and 20 mg/kg/d Fluoxetine, respectively [26] (Fig. 1A). Based on previous studies, quality control of XYS was investigated by ultra-high performance liquid chromatography (UHPLC). XYS was purchased from Changsha Jiuzhitang Co., Ltd., following the same preparation and batch number [30]. It is produced according to the production technology stipulated in the 2020 edition of the Chinese Pharmacopoeia (Chinese Pharmacopoeia Commission, 2020). FLX (CAS 56,296–78–7) was purchased from Selleck Chemicals.

### Behavioral tests

#### Sucrose preference test (SPT)

Following the methodology outlined in our previous study [31], the sucrose preference trials were executed in cages equipped with two standard drinking bottles, each containing a 1% sucrose solution (w/v). Following a 24-h period of sucrose solution consumption, the mice were provided unrestricted access to drinking water for 3 h. Subsequently, two bottles were substituted with a 1% sucrose solution and pure water. To minimize potential bias due to side preferences, the two drinking bottles were interchanged midway through the test, and the sucrose preference was then assessed.

#### Tail suspension test (TST)

Each mouse from the four groups was suspended on a horizontal rod positioned 50 cm above the ground, securing their tails with adhesive tape (1–2 cm from the base of the tail). The entire experimental session lasted 6 min, and the activity of each mouse during the final 4 min was recorded. Immobility time (seconds), indicative of the duration until the mice ceased struggling and remained motionless, was quantified. The mice were suspended 35 cm above the floor for 6 min, and their immobility over the last 4 min was measured.

#### Open field test (OFT)

The OFT was conducted in a quiet environment, ensuring that mice acclimatized to the laboratory for at least 10 min. Mice were positioned at the center of the open field reaction box, and cameras, along with timing equipment, were arranged to capture all movement trajectories within the 5-min trial. This included monitoring entries into the central area. Post-trial, thorough cleaning of the arena was performed using 75% alcohol after each experiment to eliminate any potential influence of residual odors on subsequent mice.

#### Elevated plus-maze test (EPM)

The maze comprised two closed arms (30 × 7 cm) enclosed by 20 cm baffles on each side and two open arms (30 × 7 cm), with a central platform (7 × 7 cm) at the intersection of the arms. Animals from each group were individually positioned in the central area (7 × 7 cm) of the maze. The movements of the mice were observed and recorded for 5 min.

### Bacterial DNA extraction, 16S rRNA sequencing, and analyses

Microbial DNA was extracted from colonic contents using the E.Z.N.A.® DNA Kit (Omega Bio-Tek, Norcross, GA, U.S.), according to the manufacturer’s instructions. The final DNA concentration and purity were determined using a NanoDrop 2000 UV–vis spectrophotometer (Thermo Scientific, Wilmington, USA), and DNA quality was evaluated by 1% agarose gel electrophoresis.

The V3–V4 hypervariable regions of the bacterial 16S rRNA gene were amplified with primers 341F (5′-CCTAYGGGRBGCASCAG-3′) and 806R (5′-GGACTACNNGGGTATCTAAT-3′) using a thermocycler PCR system (GeneAmp 9700, ABI, USA). The PCR products were extracted from a 2% agarose gel using the AxyPrep DNA Gel Extraction Kit (Axygen Biosciences, Union City, CA, USA) and quantified using QuantiFluor™-ST (Promega, USA), according to the manufacturer’s instructions. Diversity metrics were calculated using the core-diversity plugin within QIIME2. Feature-level α-diversity indices, such as the observed Chao1 richness estimator and Shannon diversity index, were calculated to estimate microbial diversity within individual samples. The β-diversity distance measurements, including Bray–Curtis, unweighted UniFrac, and weighted UniFrac, were performed to determine the structural variation in microbial communities across samples and then visualized by principal coordinate analysis (PCoA). The relative abundances of microbial species at different taxa levels were estimated using the R package “vegan”. The sequencing and data analysis services were provided by Wuhan Metware Biotechnology Co., Ltd. China.

### Metabolomic analyses

Metabolomics analyses were performed on microbial metabolite extracts using an ACQUITY UPLC I-Class PLUS/Xevo G2-XS QToF system (Waters Corporation, Milford, MA, USA) with a Waters Acquity UPLC HSS T3 column (2.1 × 100 mm, 1.8 μm). Samples (1 μL) were injected and eluted with a mobile phase comprising solvent A (0.1% formic acid) and B (0.1% formic acid-acetonitrile) at a flow rate of 0.4 mL/min and 40 °C column oven. The eluting gradient program was as follows: 0–0.25 min, 2.0% B; 0.25–10.00 min, 2.0–98% B; 10.00–13.00 min, 98% B; 13.0–13.10 min, 98–2.0% B; and 13.10–15.00 min, 2.0% B. The effluent was connected to an ESI-triple quadrupole linear ion trap (QQQ-LIT) mass spectrometer equipped with an ESI Turbo Ion-Spray interface operating in both positive and negative ion modes and operated using Analyst software. The ESI source operating parameters were as follows: ion source temperature, 150 °C; desolventizing gas temperature, 500 °C; capillary voltage, 2000 V; cone voltage, 30 V; cone-gas flow rate, 50 L/h; and desolventizing gas flow rate, 800 L/h. Full scans were acquired in a scan range of 50–1200 m/z with a scan frequency of 0.2 s, and data were collected by MassLynx (v. 4.2, Waters) and identified by Progenesis QI within a mass deviation of 100 ppm. The total metabolite concentration did not differ significantly between the samples. Principal component analysis (PCA) of the metabolites was performed using PAleontological STatistics software. Partial least squares-discriminant analysis (PLS-DA) was performed using SIMCA® software to improve visualization. The volcano map of total metabolites and mapping of differential metabolites via Kyoto Encyclopedia of Genes and Genomes (KEGG) enrichment analysis were drawn using the R package “ggplot2”. The PLS-DA model was assessed using Hotelling’s T2 test. The LAD score was evaluated using Kruskal–Wallis and Wilcoxon tests. The extraction, detection, and quantitative analysis of metabolites in the samples were performed by Wuhan Metware Biotechnology Co., Ltd. (www.metware.cn).

### Hematoxylin and eosin (H&E) staining

Brain tissue samples were fixed in a 10% formalin solution, underwent decalcification, dehydration, transparency establishment, and were then immersed and embedded in paraffin. Using a microtome, 10 μm-thick tissue sections were meticulously prepared. After dewaxing with xylene and transitioning through an aqueous ethanol series, the sections were subjected to H&E staining and subsequently observed under a microscope.

### AFADESI-MSI analysis

Full MS scans of AFADESI-MSI analysis were conducted in accordance with previous research [32]. Briefly, brain tissue samples were fixed, sectioned into 10 μm slices, and then stored at −80 °C. Before being subjected to AFADESI-MSI analysis, the sections underwent a thorough drying process in a vacuum desiccator for approximately 30 min. Using an AFADESI platform coupled with a quadrupole Orbitrap mass spectrometer (Q Exactive, Thermo Scientific, Bremen, Germany), AFADESI-MSI experiments were conducted with ACN:water (8:2, V/V) at a flow rate of 3 mL/min. A 3D electrical moving stage (Beijing Optical Instrument Factory, Beijing, China) exhibited a constant rate of 0.04 mm/s in the x-direction, with a 0.04-mm vertical step in the y-direction. Key mass spectrometer parameters included a capillary temperature of 350 °C, S lens voltage of 55 V, maximum injection time of 200 ms, automatic gain control target of 3 × 10^6^, resolution of 70,000, and an m/z range of 70–1000.

### Data processing and analysis

Behavioral testing utilized Behavior Analysis software, specifically the EthoVision software analysis system from Noldus Information Technology, Leesburg, VA, USA. For image reconstruction and background deduction, MassImager Pro, a mass spectrometry imaging software, was used. The entire mouse brain tissue was designated as a Region of Interest (ROI) for data extraction. The data were then imported into MarkerView 1.2.1 (AB SCIEX, USA) for peak alignment and isotope ion deletion. Subsequently, the data were normalized based on the total ion intensity [32]. The HMDB (https://hmdb.ca/) and LIPID MAPS^®^ (https://www.lipidmaps.org/) databases were consulted for metabolite identification. Supervised multivariate OPLS-DA was employed to compare the metabolic profiles among the different groups.

### Magnetic resonance imaging data acquisition, processing and analysis

A new batch of 24 mice was specifically used to obtain magnetic resonance imaging (MRI) data. These mice were randomly divided into three groups: Control group (Control), Chronic restraint stress group (CRS), XYS treatment group (CRS + XYS). All mice were quickly anaesthetized using isoflurane (Inhaled general anesthetics, Ruiwode Life Science and Technology Co., Shenzhen, China; 3% isoflurane in medical air, 2 l/min) for five minutes. Mice were then transferred to a heated imaging cradle and anesthesia was maintained with a nose cone (1.5% isoflurane in medical air, 1 l/min). An animal monitoring unit (Small Animal Instruments Inc., New York, NY, USA) was used to record the heart rate and respiratory frequency of the mice. The normal body temperature of the mice (37 °C) was maintained by a hot water circulation system.

Then, in vivo MRI experiment were all performed on a 9.4 T Bruker Biospec 94/30 USR at Jinan university (Bruker Biospin, Ettlingen, Germany). The data DTI were acquired using a SE-EPI diffusion MRI sequence. The main imaging parameters were TR = 2500 ms, TE = 18 ms, diffusion gradient duration( δ) = 2.5 ms, gradient separation (Δ) = 8.5 ms, slice thickness = 0.5 mm, matrix size = 125 × 125 × 22, spatial resolution = 0.13 × 0.13 × 0.80 mm^3^, flip angle = 90゜, average = 1, 3 b-value = 0 (b_0_) images, followed by 30 diffusion encoding gradient directions with a diffusion weighting (b) of 1200 s/mm^2^.

DTI post-processing was performed using the FMRIB software library version 6.0.2 (FSL, created by the Analysis Group, Oxford, UK). (1) DTI data were converted to NIfTi format. The skull was removed from the DTI date for each mouse. (2) The data dimension was scaled by a factor of 10 to stimulate human-similar voxel sizes to allow FSL processing. (3) The DTI data were corrected for distortions and motion artifacts. (4) Tensors were fitted using the b-factor and diffusion direction matrix with the DTIfit toolbox. The fractional anisotropy and axial diffusivity indices were calculated for each voxel resulting in diffusion weighted brain maps, including whole brain fractional anisotropy maps and axial diffusivity maps. (5) The b0 diffusion image of each mouse was coregistered to the Allen Mouse Brain template and the resulting transforming matrix was then applied to register the DTI fractional anisotropy and axial diffusivity maps. The normalized fractional anisotropy and axial diffusivity maps were smoothed using an isotropic Gaussian filter (4-mm full-width at half maximum).

Spatial-based voxel-wise statistics were also performed on the smoothed diffusion maps using two-tailed, two-sample t-tests to assess microstructure changes between the control and model groups. The threshold for statistical significance of diffusion maps were set at p < 0.005. The regions showing significant at this threshold within the medial prefrontal cortex (PFC) and hippocampus were selected as ROIs and the mean diffusion indicators in this ROI were extracted of all mice. The changes of these diffusion indicators among the three groups were detected, which can see the antidepressive effects of XYS treatment in brain microstructure within the mPFC and hippocampus. One-way ANOVA on the average fractional anisotropy and axial diffusivity values of these ROIs were calculated among the three groups. Post-hoc analyses were performed when significant differences were observed. P < 0.05 was considered statistically significant.

### Correlation analysis

Spearman correlations and redundancy analysis (RDA) were applied within each group to examine the associations between depressive behavioral parameters and metabolites in different brain regions. Selected parameters, characterized by |r|> 0.5 and *P* < 0.05, were selected for network visualization.

### Statistical analysis

The data were presented as the mean ± standard error of the mean (SEM) and analyzed utilizing SPSS 25.0 software. Initial analysis comprised repeated measures analysis of variance (ANOVA) for data demonstrating normal distribution and homogeneity of variance. For cases of non-normal distribution or heterogeneous variance, a non-parametric test of K independent samples was employed for item-by-item statistical analysis. GraphPad Prism software (Version 9.0, USA) was utilized for generating graphs, encompassing column and box plots. A significance level of *P* < 0.05 was deemed statistically significant.

## Results

### Effects of XYS on body weight and sucrose preference rates in the CRS model

Prior to the CRS experiments, no significant difference in the body weight of mice was observed among all groups (Fig S1A). Following 21 days of CRS, the body weight of mice exhibiting depressive symptoms was markedly lower than that of the control group (*p* < 0.001) (Fig. 1B). Moreover, the sucrose preference rates of mice with depressive symptoms were significantly lower than those of the control group (*p* < 0.01) (Fig. 1C). In comparison to the CRS group, both the body weight and sucrose preference rates of the CRS + XYS and CRS + FLX groups increased, but to varying extents (Fig. 1B, C).

### Effects of XYS on behaviors in the CRS model

In the TST, mice displaying depressive symptoms demonstrated a significantly increased immobility time compared to the control group (*p* < 0.05) (Fig. 1D). Remarkably, both the groups subjected to chronic restraint stress along with XYS and FLX exhibited a notable reduction in immobility time in mice when contrasted with the CRS group, indicating significant regulatory effects on behavioral despair (*p* < 0.05; *p* < 0.05).

In the OFT conducted on day 0, there were no statistically significant differences in the total distance traveled within 5 min and time spent in center zone among the four groups (Fig. S1B. C). However, following a 21-day period **(**Fig. 1E and F), mice subjected to CRS displayed a substantial decrease in both the total distance moved and the time spent in the central zone, in contrast to the control group (distance and time: *p* < 0.01), indicative of a noticeable anxiety state. Importantly, the oral administration of XYS resulted in a significant reversal of this observed change (distance: *p* < 0.01, time: *p* < 0.001).

In the EPM, the group subjected to CRS displayed a significantly reduced number of entries into the open arms compared to the control group (*p* < 0.001). The oral administration of XYS resulted in a significantly higher number of entries into the open arms compared to the CRS group, signifying a reduction in anxiety behavior in mice treated with XYS (*p* < 0.05) (Fig. 1G). Consistent with these findings, the time spent in the open arms was decreased in the CRS-induced group in comparison to the control group, while mice treated with XYS displayed an extended duration in the open arms compared to the CRS group. This suggests that the intake of XYS mitigated anxiety-like behaviors induced by CRS (Fig. 1H). Mice subjected CRS exhibited predominant movement in the peripheral area and closed arm, whereas XYS-treated mice demonstrated extended movements into the central area and open arm (Fig. 1I). The above results suggest that XYS can significantly improve anxiety-like and depressive-like behaviors in CRS mice.

### Effects of XYS on gut microbiome in the CRS model

**Fig. 2:**
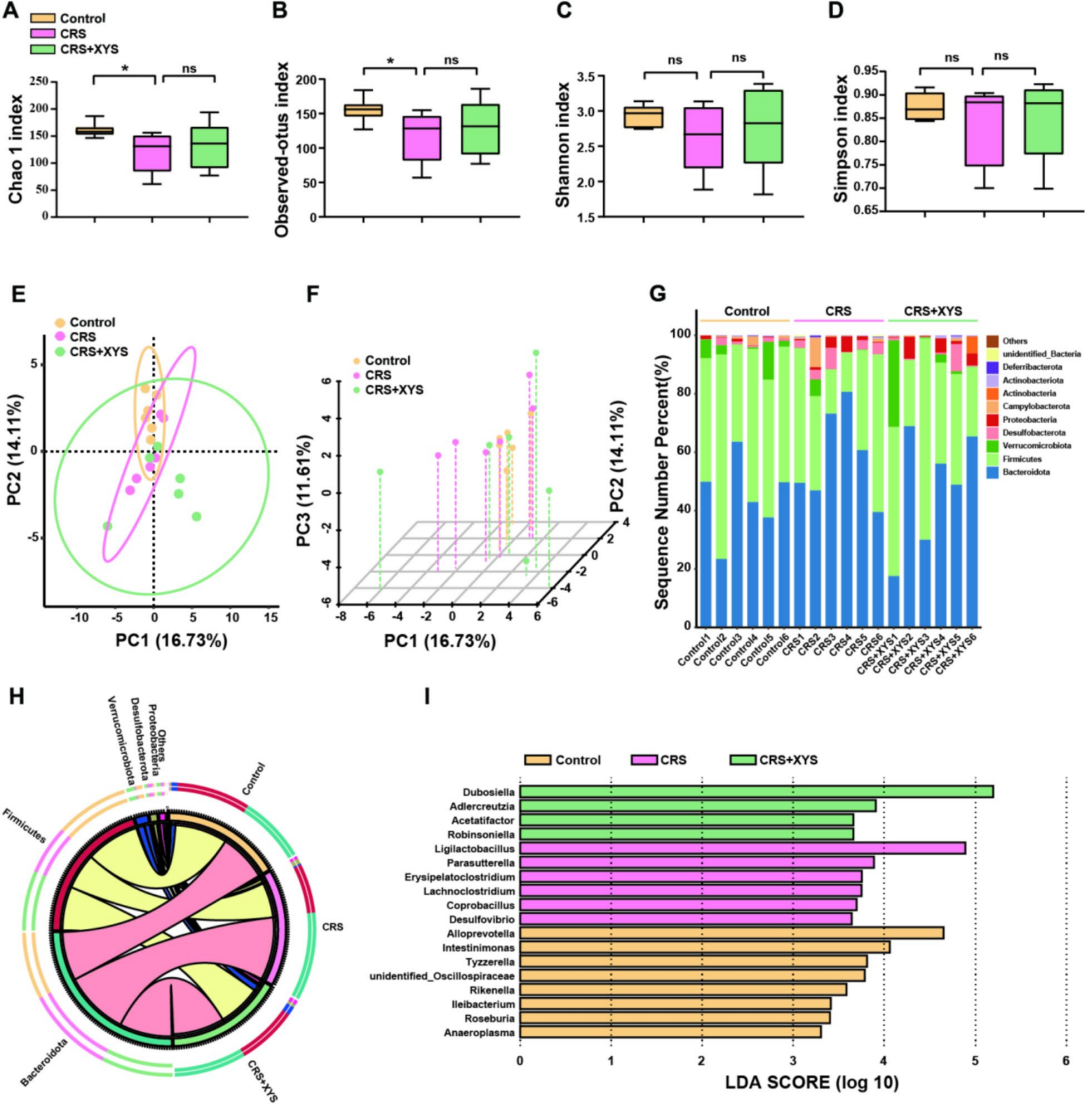
XYS rebuilds the gut microbiome of CRS-induced depressive mice. **A** α-Diversity was presented by box plot of the Chao index. **B** Observed index. **C** Shannon index. **D** Simpson index. **E**–**F** Principle coordinate analysis plot of the gut microbiota. **G**–**H** The gut microbiota composition among the experimental groups at the phylum level. **I** LEfSe. **P < 0.05 versus the control group

The microbiota composition of the colon content samples was analyzed using 16S rRNA high-throughput sequencing. Bacterial 16S rRNA raw reads were obtained from 18 colon content samples (n = 6 for each group), and 1581 distinct operational taxonomic units (OTUs) were identified. Community richness analysis showed that the Chao1 index of the CRS group was significantly lower than those of the control group (Fig. 2A). The results of species richness analysis showed that the observed index of the CRS group was significantly lower than those of the control group (Fig. 2B). In addition, the results of community diversity analysis showed that the Shannon and Simpson indices of CRS-induced group showed a downward trend compared with than those of the control group (Fig. 2C, D). Intriguingly, within the XYS group, as compared to the CRS group, there is an upward trend in the Chao1 index, the observed species richness, as well as the Shannon and Simpson indices. These results indicated that CRS treatment significantly reduced the community diversity of gut microbiota, and this effect was reversed by XYS treatment. Moreover, the results of principal coordinate analysis confirmed that the CRS group had different microbiota characteristics compared with the normal and XYS groups (Fig. 2E, F). The relative abundances of gut microbiota were enumerated at the phylum level (Fig. 2G). Firmicutes, Bacteroidetes, Verrucomicrobiota, Desulfobacterota and Proteobacteria were the dominant phyla in the fecal microbiota of the mice. Compared to the control group, CRS induction promoted a significant increase in Bacteroidetes in the CRS group and XYS intake reduced this increase (Fig. 2H). A cladogram generated by LEfSe analysis of the microbiome data (Fig. 2I) showed 6 differentially abundant clades in the CRS group and 4 differentially abundant clades at the family level in the XYS group (*p* < 0.05, LDA > 2.0).

**Fig. 3:**
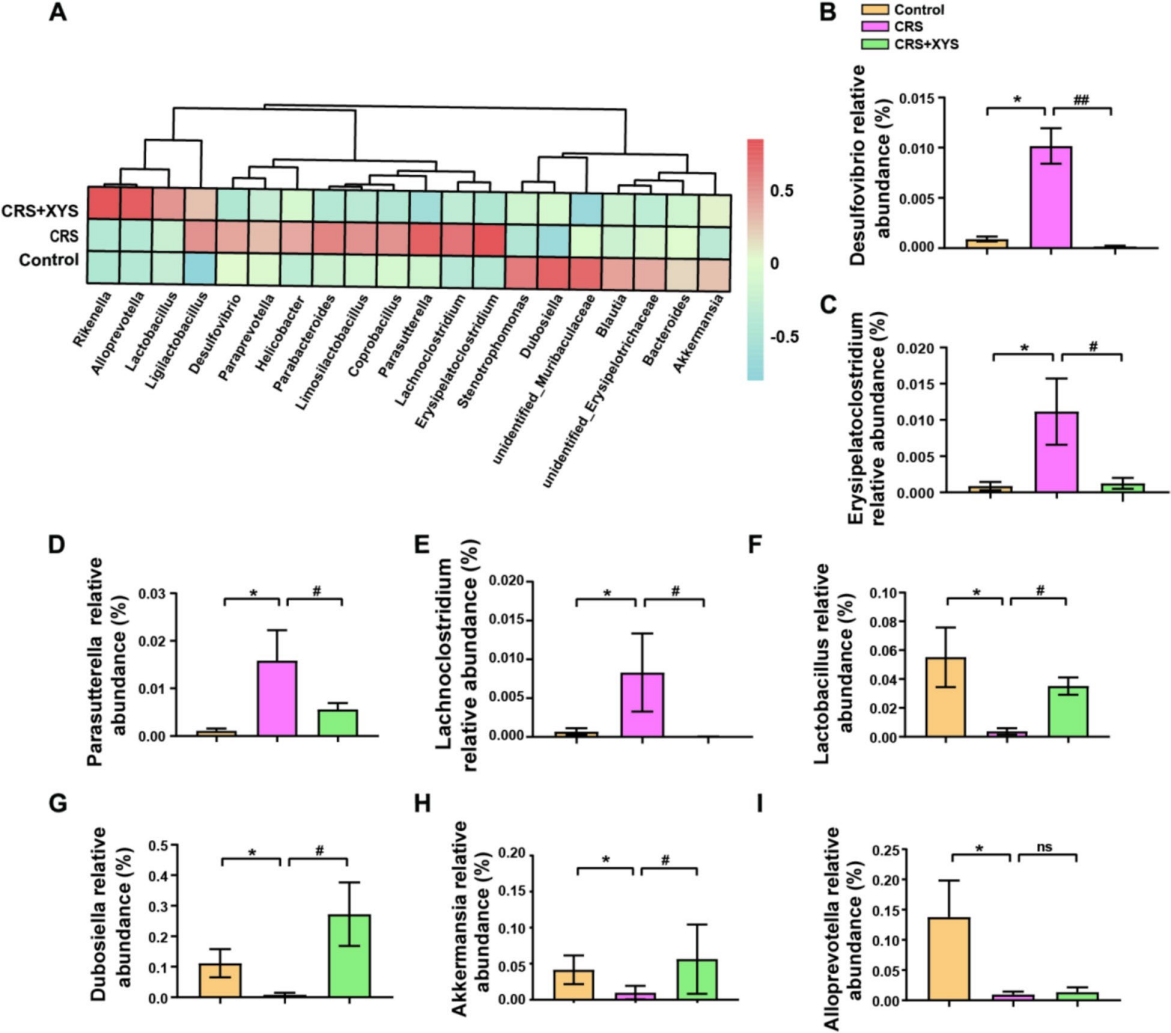
XYS rebuilds the gut microbiome of CRS-induced depressive mice. **A**–**I** Relative abundance of gut microbial community members at the genus level among the three groups. Data represent the mean ± SEM of six mice in each group. **P < 0.05 versus the control group; ^#^*P* < 0.05 and ^##^*P* < 0.01 versus the CRS group

The microbial composition was further analyzed by taxonomic profiling (Fig. 3A). At the genus level (Fig. 3B–E), compared to the control group, the relative abundances of *Desulfovibrio*, *Erysipelatoclostridium*, *Parasutterella* and *Lachnoclostridium* were significantly induced in the CRS mice, but XYS intake decreased this trend. Moreover, the abundance of *Lactobacillus* was significantly reduced in the CRS group, but XYS intake improved this decreasing trend. Moreover, oral administration of XYS significantly increased the relative abundances of *Dubosiella* and *Akkermansia*, *Alloprevotella* in mice with CRS-induced depression-like behavior (*p* < 0.01 and *p* < 0.001, respectively) (Fig. 3F–I). The above results suggest that XYS can reverse CRS-induced gut microbiota dysbiosis.

### Effects of XYS on gut microbiome metabolites in the CRS model

**Fig. 4:**
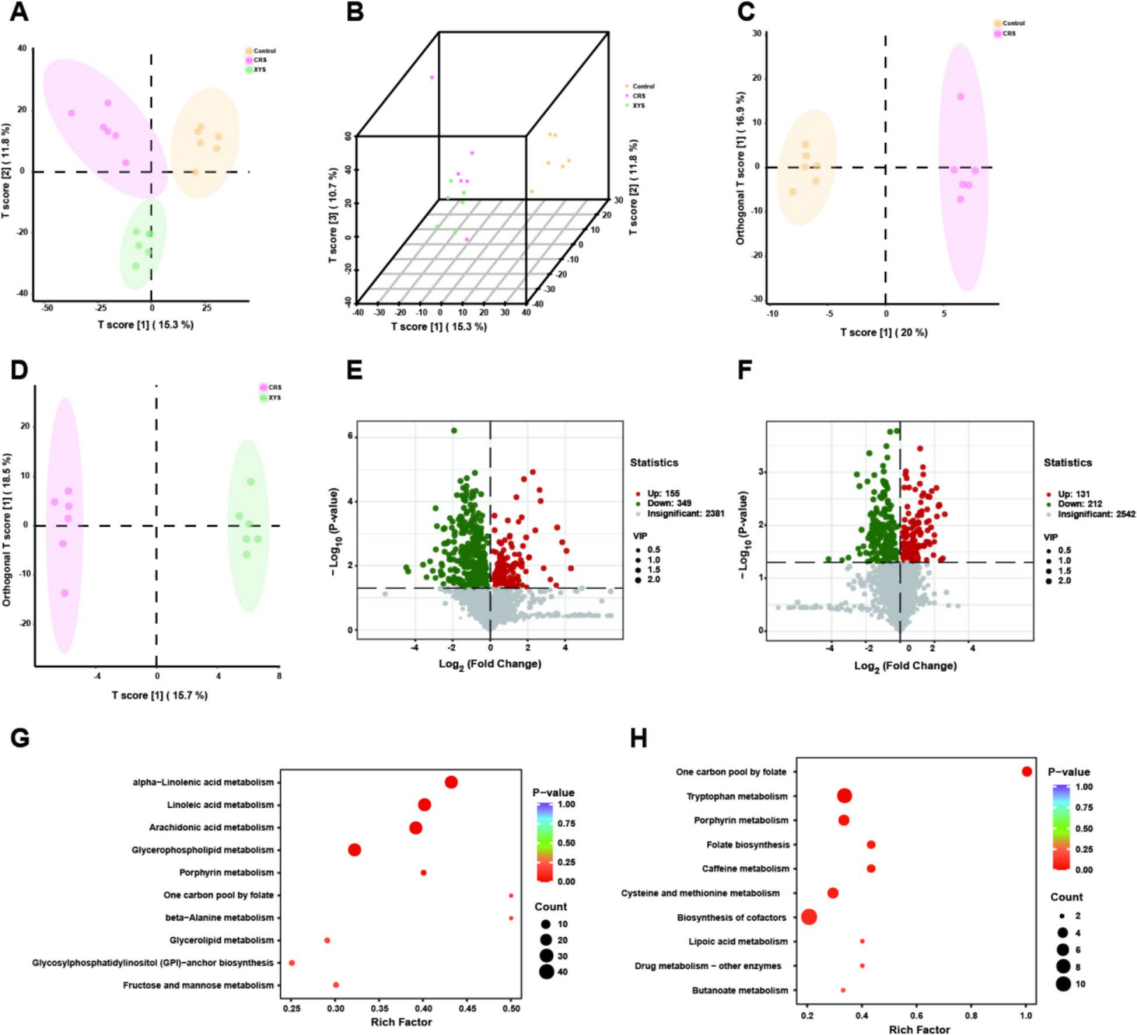
Metabolic profiles of the control, CRS and XYS groups by multivariate analysis. **A**–**B** The score scatter plots of the control, CRS and XYS groups from the PLS-DA data. **C** OPLS-DA score scatter plots for the pairwise comparisons between the control and CRS groups. **D** OPLS-DA score scatter plots for the pairwise comparisons between the CRS and XYS groups. **E** Differential analysis of gut microbial metabolites between Control and CRS groups (Volcano map). **F** Differential analysis of gut microbial metabolites between CRS and XYS groups (Volcano map). **G** Pathway topology enrichment between the control and CRS groups. **H** Pathway topology enrichment between the CRS and XYS groups. Data represent the mean ± SEM of six mice in each group

To further evaluate the effects of XYS intake on the formation of microbial metabolites, the colonic contents of the mice were analyzed using UHPLC-QTOF-MS/MS. The PLS-DA method was applied to investigate the separation of the Control, CRS and CRS + XYS groups. Separation was observed among the three groups, indicating metabolite changes in the development of depression-like behavior or ingestion of XYS (Fig. 4A, B). To further clarify these specific changes, a supervised multivariate orthogonal partial least squares-discriminant analysis (OPLS-DA) model was used to distinguish between the different variables. As illustrated by the results of the analysis, the CRS group was separated from the Control group (Fig. 4C). Notably, the XYS group was also separated from the CRS group, indicating that XYS intake improves metabolic disorders caused by CRS (Fig. 4D). In addition, a total of 2885 metabolites were identified in the comparison between the control and CRS groups, of which 155 increased and 349 decreased after CRS induction (Fig. 4E). More importantly, a total of 2885 different metabolites were identified in the comparison between the XYS and CRS groups, of which 131 increased and 212 decreased after XYS intake (Fig. 4F). KEGG topology analysis was used to identify the pathways that were enriched between the groups (Fig. 4G–H). Among them, Multiple metabolic pathways, including glycerophospholipid metabolism, linoleic acid metabolism, arachidonic acid metabolism as well as alpha-linoleic acid metabolism, were enriched between the Control and CRS groups (Fig. 4G); tryptophan metabolism and porphyrin metabolism were enriched between the CRS and XYS groups (Fig. 4H).

### XYS regulates the brain metabolite

To evaluate the impact of XYS intake on functional metabolites, we conducted AFADESI-MSI analysis on the brains of mice in both positive and negative modes. The OPLS-DA method was utilized to investigate the differentiation among the control, CRS, and CRS + XYS groups. Remarkably, a clear separation among the three groups was observed, indicating discernible metabolite alterations linked to CRS induction or XYS consumption (Fig. S2). Furthermore, 435 differential metabolites were identified the comparative analysis between the control and CRS groups. Significantly, spatial and quantitative differences were observed in lipid metabolism, tryptophan metabolism, glutamate/glutamine metabolism, acetylcholine (ACH), and adenosine metabolism between the CRS group and the CRS + XYS group. Details of the differential metabolites in the Control, CRS, and CRS + XYS groups, along with their specific distribution in brain tissue, are presented in Table 1.

**Table 1:** The differential metabolites identified in the AFADESI-MSI with brain distribution information in control, CRS and XYS-treated mice

Metabolite identification	Elemental composition	Adduct	Measured (m/z)	Tissue distribution		
				Control	CRS	CRS + XYS
PA	H3PO4	[M + K]^+^	136.9405	CTX, AM	CTX↓	CTX↑, AM↑
Phosphatidylcholine, PC (20:2/20:4)	C42H80NO8P	[M + H]^+^	834.598	HP, TH	HP↓, TH↓	HP↑, TH↑
Phosphatidylcholine, PC (20:4/14:0)	C42H76NO8P	[M + H]^+^	754.5365	HP, TH	HP↓, TH↓	HP↑, TH↑
lysophosphatidylcholine (LysoPC 20:4)	C28H50NO7P	[M + H]^+^	544.339	CTX, HP	CTX↓, HP↓	CTX↑, HP↑
Phosphatidylethanolamines, (PE 18:0/22:6)	C45H78NO8P	[M + Na]^+^	814.5374	CTX, AM	CTX↓, AM↓	CTX↑, AM↑
Phosphatidylethanolamines, (PE 20:0/18:4)	C43H78NO8P	[M + H]^+^	768.5509	CTX, AM	CTX↓, AM↓	CTX↑, AM↑
Sphingomyelin, SM (d18:0/18:4)	C41H73N6O12P	[M + K]^+^	769.5572	HP, TH, HY	HP↓, TH↓, HY↓	HP↑, TH↑, HY↑
Acetylcholine (ACh)	CH3COOCH2CH2N + (CH3) 3	[M + H]^+^	147.1254	HP, TH	HP↓, TH↓	HP↑,TH↑
Serotonin	C10H12N2O	[M + Na]^+^	199.0842	HP, TH	HP↓, TH↓	HP↑, TH↑
5-Hydroxy-L-tryptophan	C11H12N2O3	[M + H]^+^	221.0937	CTX, AM	CTX↓, AM↓	CTX↑, AM↑
Kynurenic acid	C10H7NO3	[M + NH]^+^	207.0781	CTX, AM	CTX↑, AM↑	CTX↓, AM↓
γ-Aminobutyric acid, GABA	C4H9NO2	[M + H]^+^	104.0706	TH, HY	TH↓,HY↓	TH↑, HY↑
Glutamine	C5H10N2O3	[M + H]^+^	146.0459	CTX, HP, TH, AM	CTX↑, HP, ↑TH↑, AM↑	CTX↓, HP↓, TH↓, AM↓
Glutamate	C5H9NO4	[M + H]^−^	185.0323	HP, TH	HP↑, TH↑	HP↓, TH↓
Adenosine	C10H13N5O4	[M + H]^+^	268.1040	HP, TH	HP↓, TH↓	HP↑, TH↑

(↑) Indicates significant increase in local abundance; (↓) denotes significant decrease in local abundance. *AM* amygdala, *CTX* cerebral cortex, *HP* hippocampus, *HY* hypothalamus, *TH*, thalamus. The CRS group was compared with the control group; the CRS + XYS group was compared with the CRS group

### Effects of XYS on the brain lipids

**Fig. 5:**
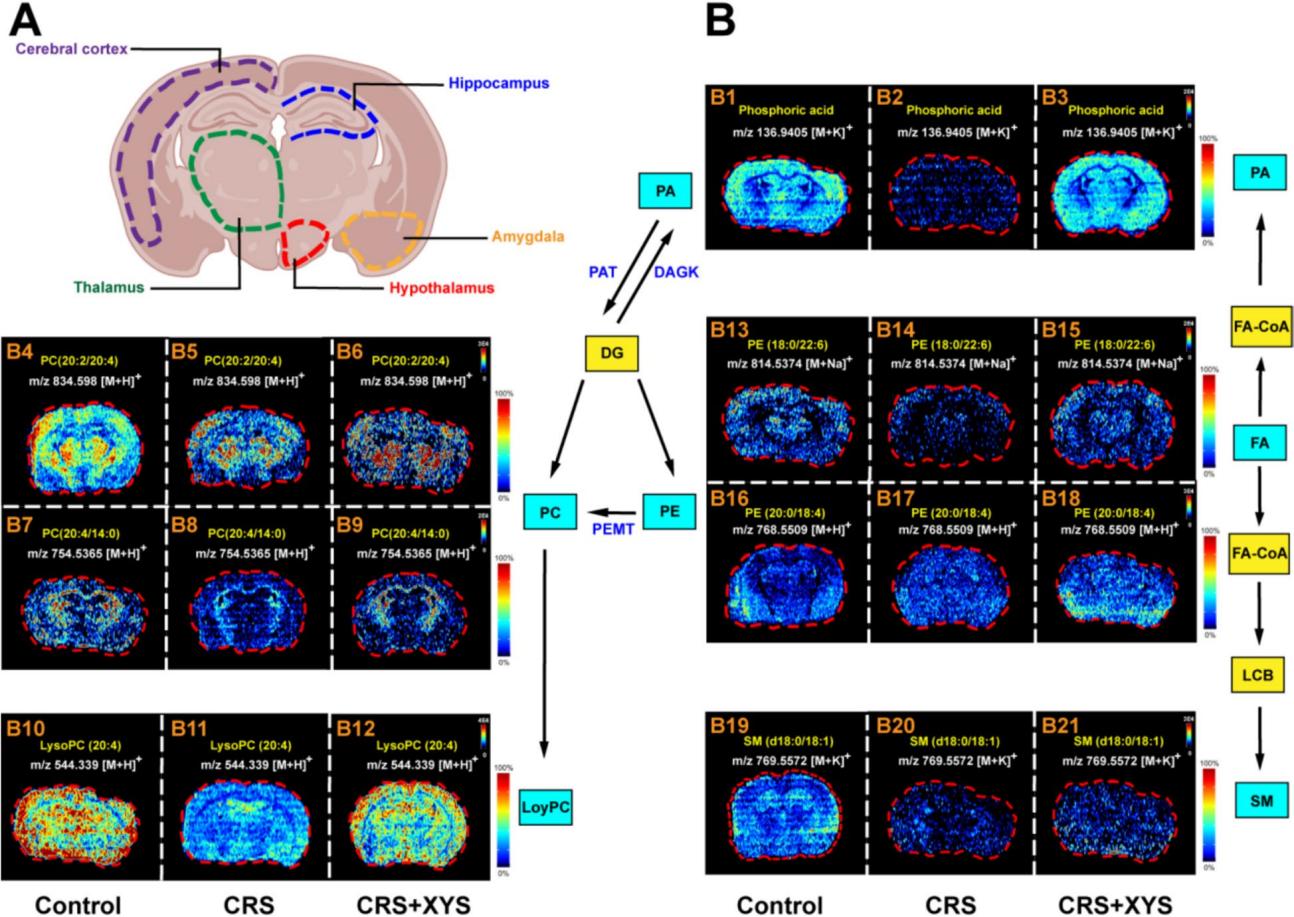
XYS significantly regulates the disorder of the spatial distribution and concentration of lipids in the brain of CRS-induced depressed mice. **A** The distribution of brain regions. **B** The spatial distribution and changes in lipids. *LysoPC*: lysophosphatidylcholine; *PA* phosphatidic acid, *PC* phosphatidylcholine, *PE* phosphatidylethanolamine, *SM* sphingomyelin

The spatial distribution and alterations in lipids are depicted in Fig. 5. Current study observed the in situ spatial distribution of a diverse range of lipids, encompassing phosphoric acid (PA), phosphatidylcholine (PC), lysophosphatidylcholine (LysoPC), phosphatidylethanolamine (PE), and sphingomyelin (SM). The distribution of brain regions is shown in Fig. 5A. Interestingly, lipids showed specific spatial distribution in brain. As shown in Fig. 5B1–B3, PA almost exclusively appeared in cerebral cortex and amygdala; PC (20:2/20:4) and PC (20:4/14:0) appeared in hippocampus and thalamus **(**Fig. 5B4–B9); LysoPC (20:4) appeared in cerebral cortex and hippocampus (Fig. 5B10–B12); PE almost appeared in cerebral cortex and amygdala (Fig. 5B13–B18); SM (d18:0/18:4) appeared in hippocampus, thalamus and hypothalamus (Fig. 5B19–B21). More importantly, regulation of lipid distribution by XYS treatment was also observed. Compared with the control group, the levels of PA, PC (20:2/20:4), PC (20:4/14:0), LysoPC (20:4), PE (18:0/22:6), PE (20:0/18:4) and SM (d18:0/18:4) were significantly decreased in CRS-induced mice (Fig. 5B). Compared with the CRS group, administration of XYS could increase the levels of PA, PC (20:2/20:4), PC (20:4/14:0), LysoPC (20:4), PE (18:0/22:6), PE (20:0/18:4) and SM (d18:0/18:4) (Fig. 5B).

### Effects of XYS on neurotransmitters

**Fig. 6:**
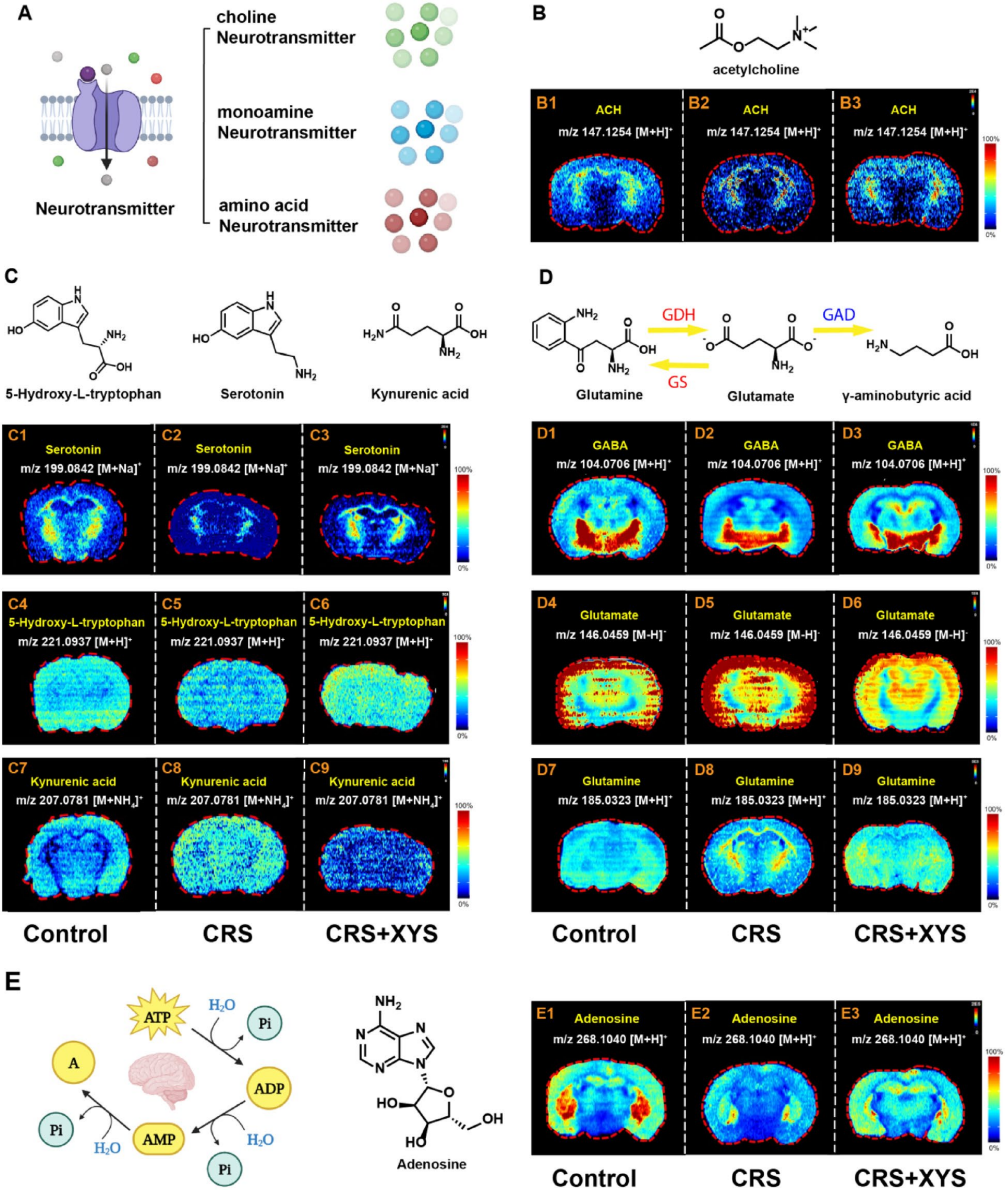
XYS significantly regulates the disorder of the spatial distribution and concentration of neurotransmitter and energy metabolites in the brain of CRS-induced depressed mice. **A** Types of neurotransmitters. **B** The spatial distribution and concentration changes of ACH. **C** The spatial distribution and changes in tryptophan metabolism. **D** The spatial distribution and changes in glutamate/glutamine metabolism. *ACH* acetylcholine, *GABA* γ-aminobutyric acid. **E** The spatial distribution and concentration changes of adenosine

The spatial distribution of neurotransmitters, including choline neurotransmitters, monoamine neurotransmitters, and amino acid neurotransmitters, were identified, involving ACH, tryptophan, glutamate/glutamine metabolism and other metabolic pathways (Fig. 6A). The spatial concentration changes of ACH was shown in Fig. 6B. As shown in Fig. 6B1–B3 ACH was mainly distributed in the hippocampus and thalamus. In comparison to the control group, the abundance of ACH were significantly decreased in mice induced with CRS. Compared with the CRS group, XYS treatment could significantly increase the concentration of ACH.

The spatial distribution and changes in tryptophan metabolism are shown in Fig. 6C. Similar to lipid distribution, serotonin, 5-Hydroxy-L-tryptophan and kynurenic acid also showed specific spatial distribution in brain. As shown in Fig. 6C1–C3, serotonin almost exclusively appeared in hippocampus and thalamus. While, 5-Hydroxy-L-tryptophan and kynurenic acid mainly appeared in cerebral cortex and amygdala (Fig. 6C4–C9). In addition, the distribution of serotonin, 5-Hydroxy-L-tryptophan and kynurenic acid in the brain were also observed. In contrast, XYS treatment significantly elevated the concentration of serotonin and 5-Hydroxy-L-tryptophan compared to the CRS group (Fig. 6C1–C6). Additionally, an increased concentration of kynurenic acid was observed in CRS mice compared with the normal group, and XYS treatment effectively reduced the concentration of kynurenic acid (Fig. 6C7–C9).

The spatial distribution in glutamate/glutamine metabolism of brain are illustrated in Fig. 6D. Similarly, glutamine, glutamate and γ-aminobutyric acid (GABA) showed specific spatial distribution in brain. As shown in Fig. 6D1–D3, GABA mainly appeared in thalamus and hypothalamus; glutamate appeared in cerebral cortex, hippocampus, amygdala and thalamus (Fig. 6D4–D6); glutamine mainly appeared in hippocampus and thalamus (Fig. 6D7–D9). The distributional difference of glutamate, glutamine, and GABA in the brain were also observed. In contrast to the control group, the concentration of glutamate was markedly elevated in mice induced with CRS. Compared with the CRS group, XYS treatment could significantly decrease the enrichment of glutamate. Besides, the decreased concentration of glutamine and GABA were observed in CRS mice compared with the normal group, XYS treatment could increase the concentration of glutamine and GABA.

### Effects of XYS on energy metabolites

We explored the changes in the spatial distribution of metabolites related to energy metabolism in each group of mice. The spatial content changes of adenosine was also observed after CRS and XYS intervention. Adenosine is a product of ATP degradation (Fig. 6E). As shown in Fig. 6E1–E3, adenosine was mainly distributed in the hippocampus and thalamus. In comparison to the control group, the abundance of adenosine was notably reduced in mice induced with CRS. However, following XYS treatment, there was a significant increase in the concentration of adenosine compared to the CRS group.

### Effects of XYS on the brain diffusion properties

**Fig. 7:**
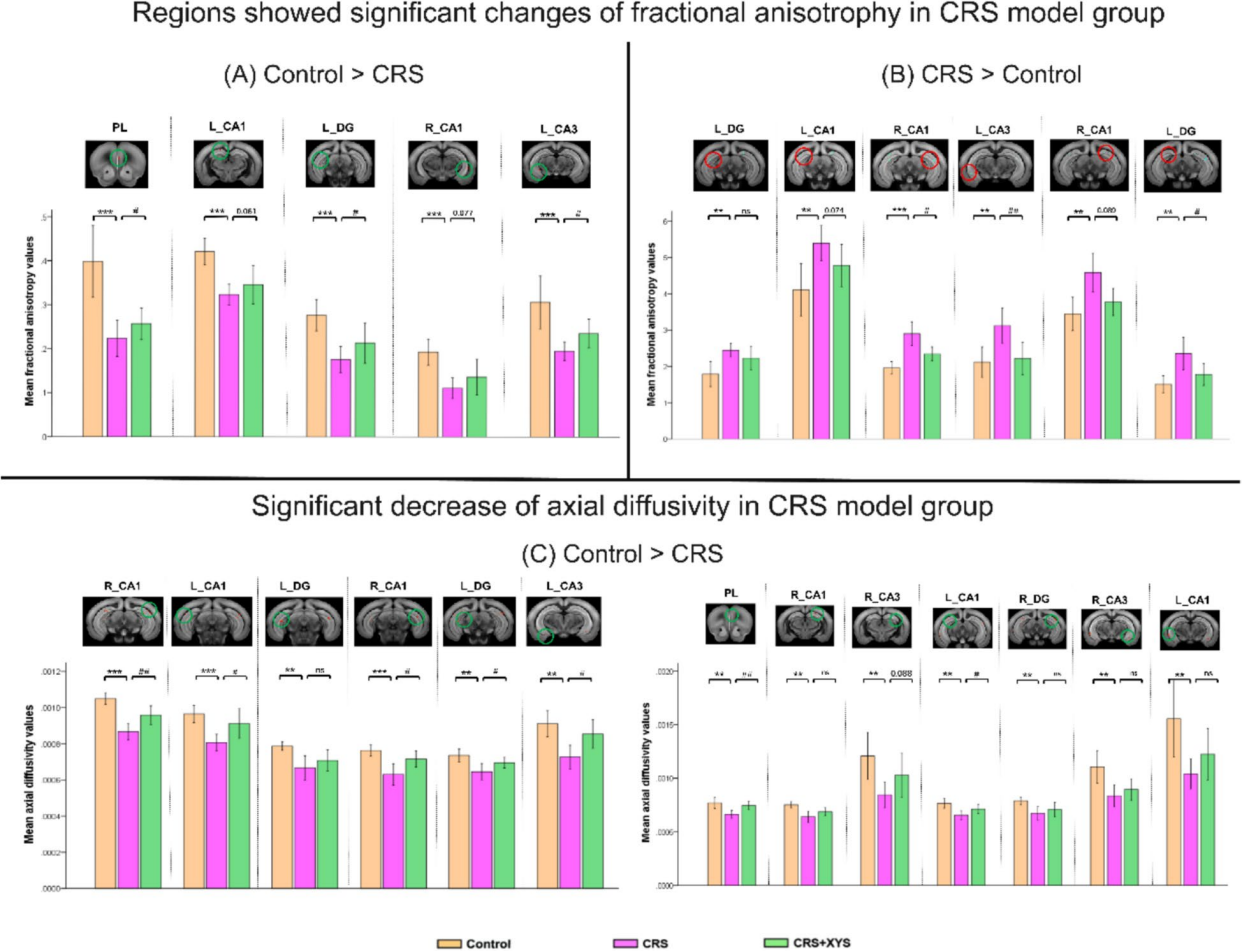
Significant changes of the diffusion indicators were observed in the prefrontal cortex and the hippocampus regions between the model group and the normal control group. **A** Regions showed significant decrease of the fractional anisotropy in the CRS model group. XYS significantly enhanced the fractional anisotrophy values and the results were presented in bar chart. **B** Regions showed significant increase of the fractional anisotropy in the CRS model group. XYS significantly depressed inhibited the fractional anisotrophy values and the results were presented in bar chart. **C** Regions showed significant decrease of the axial diffusivity in the CRS model group. XYS significantly enhanced the axial diffusivity values and the results were presented in bar chart

We conducted voxel-based DTI analysis to investigate the impact of chronic restraint stress on brain diffusion properties in depressive-like mice. As illustrated in Fig. 7A and B, compared to the control group, the fractional anisotropy values in the model group were significantly reduced in the prelimbic area of mPFC. In the regions of hippocampus, we observed that the model group showed diverse changes. Some sub-regions exhibited a significant increase of the fractional anisotropy, while others showed a significant decrease of the fractional anisotropy. As illustrated in Fig. 7C, compared to the control group, the axial diffusivity indicators in the model group consistently showed significant decreases in both the hippocampus and the prelimbic area of mPFC.

### Correlation analysis among the gut microbiome, the brain metabolites and behavior

**Fig. 8:**
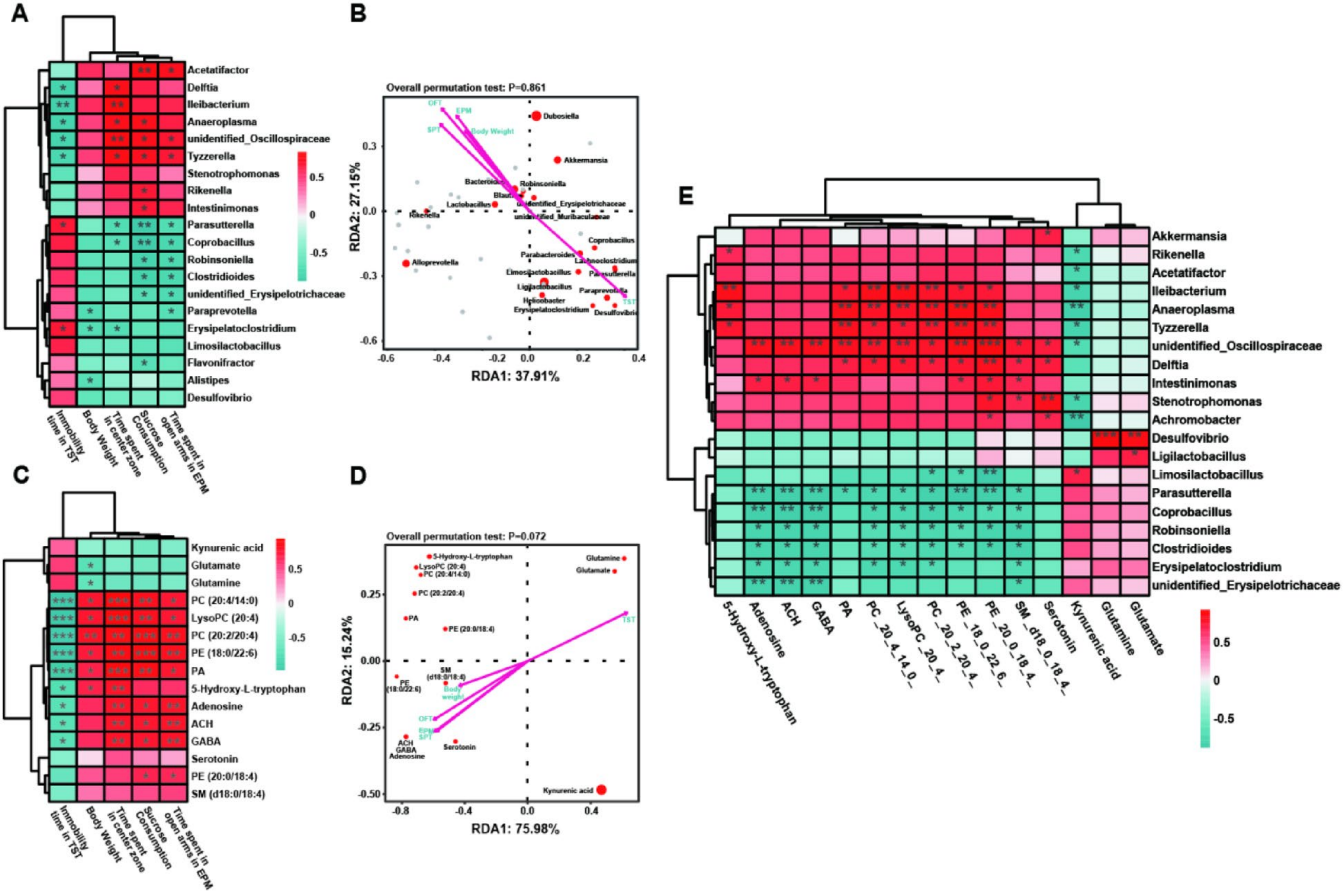
Correlation analysis among the gut microbiome, the brain metabolites and behavior. **A** Spearman’s correlation between gut microbiome and behavioral abnormalities. **B** RDA analysis between gut microbiome and behavioral abnormalities. **C** Spearman’s correlation between brain metabolites and behavioral abnormalities. **D** RDA analysis between brain metabolites and behavioral abnormalities. **E** Spearman’s correlation between brain metabolites and gut microbiome

To investigate the potential relationship among gut microbiome, brain metabolites and behavior, we conducted Pearson correlation analysis of gut microbiome, brain metabolites and behavioral abnormalities (including body weight, immobility time in TST, time spent in center zone in OFT, and time spent in open-arm in EPM) in CRS-induced depressive mice (Fig. 8). Firstly, we explored the relationship between gut microbiota and behavioral abnormalities (Fig. 8A). The results revealed that the abundance of *Parasutterella* were positively correlated with immobility time in TST, but negatively correlated with body weight, central area residence time in OFT and residence time in EPM, indicating that the increase of *Parasutterella* was associated with the onset of depression-like behavior. Moreover, the results based RDA analysis also showed that the behavioral results of immobility time in TST strongly correlated with the abundance of *Parasutterella* (Fig. 8B). In addition, the results revealed that the abundance of *Tyzzerella*, *Anaeroplasma* and *unidentified_Oscillospiraceae* were positively correlated with body weight, central area residence time in OFT and residence time in EPM, but negatively correlated with immobility time in TST.

Secondly, we conducted Pearson correlation analysis of brain metabolites and behavioral abnormalities. The results revealed that the concentration of PC (20:4/14:0), LysoPC (20:4), PC (20:2/20:4), PE (18:0/22:6), PA, 5-Hydroxy-L-tryptophan, adenosine, ACH, GABA were positively correlated with body weight, central area residence time in OFT and residence time in EPM, but negatively correlated with immobility time in TST, indicating that the increase of PC (20:4/14:0), LysoPC (20:4), PC (20:2/20:4), PE (18:0/22:6), PA, 5-Hydroxy-L-tryptophan, adenosine, ACH, and GABA was associated with the improvement of depression-like behavior. Besides, we also observed that the abundance of glutamate and glutamine was negatively correlated with body weight, indicating that the disorder of glutamate/glutamine metabolism may be related to the reduction of body weight (Fig. 8C). What’s more, RDA analysis has also been used to correlate brain metabolites with behavior (Fig. 8D). The results showed that the behavioral results of body weight, SPT, OFT and EPM were strongly correlated with the concentration of ACH, GABA, adenosine, serotonin, PE (18:0/22:6) and SM (d18:0/18:4). In addition, the immobile time in TST was strongly correlated with glutamate and glutamine.

More importantly, we conducted Pearson correlation analysis of gut microbiota and brain metabolites. The results revealed that the abundance of *Parasutterella, Coprobacillus, Robinsoniella, Clostridioides* and *Erysipelatoclostridium* were negatively correlated with SM (d10:0/18:4), LysoPC (20:4), PC (20:2/20:4), PC (20:4/14:0), PE (18:0/22:6), PA, GABA, ACH and adenosine, indicating that the change in the abundance of gut microbiota may affect the expression of brain functional metabolites. Besides, the positively correlation between the abundance of *Limosilactobacillus* and the expression of kynurenic acid was observed. Moreover, the results also revealed that the abundance of *Ileibacterium, Anaeroplasma, Tyzzerella,* and *unidentified_Oscillospiraceae* were positively correlated with SM (d10:0/18:4), LysoPC (20:4), PC (20:2/20:4), PC (20:4/14:0), PE (18:0/22:6), PA, GABA, ACH and adenosine (Fig. 8E).

## Discussion

In this study, we investigated the gut microbiome, gut microbiome metabolites and endogenous metabolic alterations in the brain of the CRS depression model, focusing specifically on the microstructural changes of prefrontal cortex and hippocampal regions in depression. Our results revealed that CRS mice exhibited pronounced depression-like behaviors, accompanied by an imbalance in intestinal microbiota, disturbances in microbial metabolites, and metabolic dysregulation in the brain. Treatment with XYS significantly alleviated depressive-like behaviors by restoring gut microbiota balance and mitigating metabolic disruptions in the gut-brain axis. Notably, the antidepressant effects of XYS were linked to the modulation of gut microbiota composition and metabolic pathways, including lipid metabolism, ACH, tryptophan metabolism, glutamate/glutamine metabolism, and adenosine.

### Regulatory effect of XYS on the imbalance of gut microbiota

The imbalance of intestinal flora is a key pathological mechanism of depression. Studies have shown that changes in gut microbiota are observed in patients with depression, and probiotic interventions can alleviate depressive symptoms [33]. Besides, antidepressants have been shown to attenuate gut microbial differences between depressed and healthy individuals, highlighting the potential role for gut microbiota as a predictor of treatment response [34]. Our prior studies have reported that orally administered XYS alleviated depressive symptoms through the improvement of gut microbiota [24, 26, 35]. In CRS-induced depression, we found that the oral administration of XYS could decrease the relative abundances of *Desulfovibrio*, *Erysipelatoclostridium*, *Parasutterella*, and *Lachnoclostridium*. *Desulfovibrio* belongs to the phylum Proteobacteria, which is implicated in inflammation and has been reported to be abundant in the gut microbiota of patients with depression and irritable bowel syndrome [36]. *Parasutterella* is a gram-negative, symbiotic bacterium that establishes long-term colonization within the human gut, occupying distinct intestinal niches. It exerts influences on host metabolism and has been observed to exhibit increased abundance in individuals with obesity, type 2 diabetes, anxiety, depression, as well as inflammatory bowel disease [37]. In this study, XYS was porved to significantly increase the relative abundances of *Dubosiella*, *Akkermansia*, and *Alloprevotella* in mice with CRS-induced depression-like behavior. *Dubosiella*, a bacterium known for its significant production of short-chain fatty acids, has been reported to exhibit anti-inflammatory effects through the modulation of the Treg/Th17 immune response and the enhancement of mucosal barrier integrity [38]. In conclusion, CRS can induce gut microbiota dysbiosis by increasing the abundance of opportunistic pathogenic bacteria and reducing the abundance of probiotics. In contrast, XYS can reverse CRS-induced gut microbiota dysbiosis by restoring microbiota richness and diversity, reshaping the overall structure of the microbiota, and regulating the balance between pro-inflammatory and beneficial genera. This finding provides mechanistic support at the gut microecological level for the antidepressant effect of XYS.

### Regulatory effect of XYS on the disturbance of microbial metabolites

The biosynthesis and metabolic transport of functional metabolites, including neurotransmitters, amino acids, and lipids, are intimately associated with intestinal microbiota and their enzymatic activities [8, 9]. In this study, UHPLC-QTOF-MS/MS was employed to investigate whether XYS altered the landscape of microbial metabolism in mice with depression. Our results showed that the microbial metabolites affected by CRS or XYS were mainly involved in glycerophospholipid metabolism, tryptophan metabolism and porphyrin metabolism. Disruption of glycerophospholipid metabolism is recognized as a critical pathological mechanisms of depression [39]. A study involving 41 patients identified alterations in glycerophospholipid metabolism as potential biomarkers of depression during early pregnancy [40]. In the current study, CRS-induced stress significantly disrupted glycerophospholipid metabolism mediated by the gut microbiota. Interestingly, changes in lipid profiles were also detected in the brain, suggesting that modifications in phospholipids may constitute pivotal elements of the microbially regulated metabolic interplay between gut and brain. Tryptophan, an essential amino acid, plays a pivotal role in immune responses, growth, development, and emotional behavior [41]. Consequently, it has emerged as a therapeutic target for a multitude of diseases, including tumors, autoimmune disorders, and neurological conditions. Tryptophan undergoes three metabolic pathways: the kynurenine metabolism pathway, serotonin metabolism pathway, and the aryl hydrocarbon receptor metabolism pathway, all of which are regulated by gut microbiota [42]. In this study, we found that the depression model exhibited significant disturbances in tryptophan metabolism. XYS significantly regulated tryptophan metabolic profiles in both gut and brain, suggesting the critical role of tryptophan metabolism in the occurrence of depression and the antidepressant efficacy of XYS.

### Regulatory effect of XYS on dysregulation of lipid metabolism in brain

Lipids are fundamental components of the brain, playing integral roles in neuroendocrine function, signal transduction and energy reserves. These roles collectively influence biological processes related to cognition, emotional states, and memory [43, 44]. The structural diversity of brain lipids spans various classes, including phospholipids, sphingolipids, and triglycerides, with phosphatidylethanolamine (PE) emerging as the most abundant, followed by phosphatidylcholine (PC), phosphatidic acid (PA), and sphingomyelin (SM) [45]. Emerging evidence suggests a connection between depression and lipid dysregulation, particularly in phospholipids and sphingolipids. Oliveira et al. identified significant reductions in PE and SM levels, specifically in the prefrontal cortex, indicating spatially specific lipid alterations associated with depression onset [46].

In this study, we explored the spatial distribution and concentration of brain lipids, revealing distinct spatial patterns. Notably, PA and PE were predominantly found in the cerebral cortex and amygdala, PC in the hippocampus and thalamus, LysoPC in the cerebral cortex and hippocampus, and SM in the hippocampus, thalamus, and hypothalamus (Fig. 5). Alterations in lipid concentration correlated with the emergence and amelioration of depression-like behaviors. We observed a decrease in the levels of PA, PC, LysoPC, PE, and SM in the brains of mice with depression-like behavior.

After treatment with XYS, the concentration of PA, PC, LysoPC, PE and SM were decreased. Given the abundance of PE in the brain, its reported modulation of ATP production, glucose metabolism, involvement in nerve cell metabolism, and synaptic signaling underscore its pivotal role [47, 48]. Consistent with previous researches, we identified reduced concentrations of multiple PE species, including PE(18:0/22:6) and PE(20:0/18:4), in the prefrontal cortex of depressed mice [46]. XYS treatment led to a substantial increase in PE level, aligning with observed improvements in depression-like behavior.

Additionally, CRS-induced depression resulted in reductions in PA, PC, and LysoPC in the brain. Notably, studies on PC levels during depression have reported varying outcomes. For instance, a mouse model of chronic unpredictable stress (CUS)-induced depression exhibited elevated PC levels [49], while another study demonstrated increased levels of specific PC species, such as PC(30:0) and PC(32:0), following treatment with the antidepressant maprotiline [50]. These findings highlight the complexity of PC metabolism in depression, suggesting that individual PC species and their responses to interventions play distinct roles in lipid dysregulation.

SM, a critical component in neuroplasticity and neuronutrition, is essential for signal transduction pathways [51]. In our study, decreased concentration of SM (d18:0/18:4) in the hippocampus, thalamus, and hypothalamus of CRS mice indicateddisrupted SM metabolism linked to changes in neuronutrition and plasticity, inducing the generation of depression-like behavior. XYS treatment significantly increased the level of SM (d18:0/18:4) in these brain regions, suggesting its regulatory effect on lipid metabolism disorder associated with depression. These results suggest the pathological features of brain lipid metabolism dysregulation in CRS-induced depressed mice and highlight the therapeutic potential of XYS in modulating lipid alterations associated with depression.

### Regulatory effect of XYS on dysregulation of neurotransmitter metabolism

Neurotransmitters are vital messengers in synaptic transmission, facilitating information flow within the CNS. They play a pivotal role in the onset and progression of depression. Neurotransmitters can be broadly classified into choline neurotransmitters, monoamine neurotransmitters, and amino acid neurotransmitters. Acetylcholine (ACH) is the primary choline neurotransmitter in the brain, implicated in learning, memory, mood regulation, and the onset of both anxiety and depression. The homeostasis imbalance of ACH in the brain induced the development of depression [52]. Reduced ACH levels can impair learning, memory, and mood, whereas overactivation of ACH and its receptor (acetylcholinesterase) induces oxidative stress, contributing to depression-like behaviors [52]. Scopolamine, a non-selective antagonist of the muscarinic acetylcholine receptor, has demonstrated efficacy in improving depressive symptoms in patients and rodent models [53, 54]. In this study, CRS-induced mice exhibited decreased ACH levels, particularly in the cortex. Treatment with XYS restored cortical ACH levels, indicating that reduced ACH, rather than overactivation, may underlie CRS-induced depressive behaviors.

Serotonin (5-hydroxytryptamine) is a primary monoamine neurotransmitter associated with depression onset and progression [7]. Serotonin synthesis is driven by tryptophan metabolism, which includes the tryptophan-5-hydroxytryptophan and tryptophan-kynurenine pathways. Depression is characterized by decreased serotonin and increased kynurenine levels, highlighting a serotonin/kynurenine imbalance as a key pathological feature [55]. In this study, serotonin was predominantly localized in the hippocampus and thalamus, while its precursor, 5-hydroxy-L-tryptophan, was mainly found in the cerebral cortex and amygdala. CRS-induced mice exhibited decreased serotonin and 5-hydroxy-L-tryptophan levels, which were significantly restored following XYS treatment. These findings align with previous studies demonstrating XYS’s modulation of tryptophan metabolism, including regulation of TPH2 and IDO1 levels [28]. Additionally, kynurenic acid, a metabolite of the kynurenine pathway, was elevated in the cerebral cortex and amygdala of CRS mice. Elevated kynurenic acid has been associated with neuroinflammation and depression [56, 57]. XYS treatment reduced kynurenic acid levels, further supporting its role in correcting tryptophan metabolism disorders and alleviating depressive symptoms.

The balance between excitatory neurotransmitter glutamate and inhibitory neurotransmitter GABA is critical for maintaining neuronal function. The glutamate-glutamine cycle in astrocytes is essential for maintaining glutamate homeostasis, with glutamate and glutamine interconverted and glutamine converted into GABA by glutamate dehydrogenase. Disruption of this cycle leads to depression [58]. Elevated glutamate levels in cerebrospinal fluid and brain tissue have been observed in depression, often accompanied by neurotoxicity caused by persistent glutamate accumulation [59, 60]. In current study, CRS mice exhibited increased glutamate and glutamine levels in the hippocampus and thalamus, alongside decreased GABA levels in the thalamus and hypothalamus, confirming glutamate-glutamine/GABA cycle disruptions in depression. XYS treatment reduced glutamate and glutamine levels while increasing GABA levels in the affected regions, suggesting that its antidepressant effects may involve modulation of these imbalances. Previous research has shown that XYS exerts antidepressant-like effects by mitigating glutamate-induced neuronal damage in the frontal cortex [61].

### Regulatory effect of XYS on adenosine metabolism dysregulation in the brain

Adenosine is a product of the breakdown of adenosine monophosphate (AMP). In the brain, adenosine triphosphate (ATP), adenosine diphosphate (ADP), and cAMP could all be degraded into adenosine. In 2004, Kaster et al. demonstrated that adenosine could alleviated depressive symptoms in mice, revealing the association between adenosine and depression [62]. The possible antidepressant mechanism of adenosine is related to energy metabolism, levels of brain-derived neurotrophic factor and GABA, which were both mediated by adenosine receptors A1R and A_2A_R. In particular, the activation of A_2A_R could promote the accumulation of BDNF and GABA, thereby alleviating depression [63]. A previous study showed that the pathological manifestations of increased A_2A_R activity and excessive activation of microglia exist in CRS-induced depression rats, while XYS could reduce A_2A_R activity and maintain adenosine concentration to exert antidepressant effects [64]. In the current study, we observed a decrease concentration of adenosine in hippocampus and thalamus of CRS mice, while XYS intervention could increase the level of adenosine, further enriching the evidence that XYS regulates adenosine to alleviate depression.

### Regulatory effect of XYS on metabolism in the prefrontal cortex and hippocampus

The prefrontal cortex and hippocampus are key brain regions implicated in depressive disorders. The hippocampus is primarily associated with memory storage, learning, and emotional regulation, while the prefrontal cortex plays a vital role in functions like olfaction, attention, emotion, and memory processing. In the depression model induced by CRS, the disturbance in PC (20:2/20:4), PC (20:4/14:0), LysoPC (20:4), SM (d18:0/18:4), ACH, serotonin, glutamate, glutamine, and adenosine was predominantly observed in the hippocampus. This indicates that the metabolic disorder in the hippocampus plays a crucial role in the pathogenesis of depression. Notably, XYS demonstrated the ability to alleviate depression by regulating lipid and neurotransmitter metabolism in the hippocampus, underscoring the importance of hippocampal regulation for the antidepressant effects of XYS.

Moreover, alterations in the prefrontal cortex of the CRS-induced depression model, such as increased the concentration of kynurenic acid and glutamate and decreased the concentration of 5-Hydroxy-L-tryptophan, were observed. XYS was found to enhance the level of 5-Hydroxy-L-tryptophan while inhibiting the accumulation of kynurenic acid and glutamate in the prefrontal cortex. This reflects the compound’s regulatory effect on neurotransmitters, highlighting the prefrontal cortex as another crucial regulatory region for XYS.

### Regulatory effect of XYS on brain diffusion properties in the prefrontal cortex and hippocampus

The pathway from the ventral hippocampus to the mPFC is thought to play a significant role in emotional memory processing. Therefore, the information transmission on this pathway was considered to be disrupted in the depressive state, which could be related to its impaired synaptic plasticity. The analysis results of the present study found that both fractional anisotropy and the axial diffusivity in the prelimbic area of mPFC and some subregion of hippocampus were significantly reduced in the model group. A decreased axial diffusivity value usually indicates axon injury, such as axon atrophy, fracture or microstructure disorder. Fractional anisotropy, which represents the directionality of water diffusion, is the most commonly used measure of the anisotropic component of DTI, and it is a highly sensitive marker of white matter integrity. A decreased fractional anisotropy value usually indicates the decreased white matter integrity of demyelination. The changes in the diffusion tensor coefficients in the two brain regions of the CRS model group were consistent with the previous studies in depression [38, 65, 66]. The decreased fractional anisotropy with decreased axial diffusivity in these region observed in this study suggests that chronic stress may damage the myelin integrity of brain areas related to depression. Damage to white matter integrity in the prefrontal-hippocampus regions may influence mental activity and disturb regulation of the emotional arousal network, which has been proven to play an important role in depression patients [67]. In the XYS intervention group, the significant reduction in these diffusion indicators was reversed. This tendency is consistent with the results of our lipid metabolism and behavioral analysis, demonstrating that XYS intervention can reverse the stress-induced neuroplasticity alterations in the prefrontal cortex and hippocampus. Taking into account the relationship between neurotransmitter changes and brain tissue lipid metabolism [68, 69], as well as the potential impact of neurotransmitter changes on the microstructure and function of the brain [7], we infer that there is a correlation between brain tissue lipid metabolism and the corresponding brain structure and function. Our study revealed abnormalities in lipid metabolism (e.g., PC, SM, PA) and significant alterations in diffusion metrics reflecting myelin integrity within brain regions including the prefrontal cortex and hippocampus. The spatial congruence observed between these metabolic changes and microstructural alterations suggests potential impacts of lipid dysregulation on regional neural architecture. However, current findings are insufficient to directly elucidate the interaction mechanisms between these two dimensions, necessitating specifically designed experiments in future investigations to validate their causal relationship. These MRI findings further corroborate the established correlation between gut metabolism and cerebral lipid metabolism in the present study, offering neuroimaging evidence of microstructural alterations that substantiates the brain-gut axis theory.

In hippocampus, we found that the model group not only had a decrease of fractional anisotropy but also an increase of fractional anisotropy in some subregions. Although reduced fractional anisotropy in brain regions is a common finding in depression, study have also reported that patients exhibiting depressive symptoms or Huntington’s disease also show decreased fractional anisotropy values in brain areas [70, 71]. Potential factors causing an increase in fractional anisotropy values under disease conditions mainly include: gliosis leading to increased structural rigidity, compensatory myelin remodeling temporarily enhancing directional diffusion, and apoptosis of specific neuron subgroups causing alignment of remaining axons, all of which can result in an increase in fractional anisotropy values [72, 73]. In the current study design, we cannot confirm which specific factor causes the increase in fractional anisotropy values in these brain regions, and further experiments need to be designed for verification in the future. Additionally, we observed that areas with increased fractional anisotropy values were also adjacent to areas with decreased fractional anisotropy values, suggesting that the bidirectional changes in fractional anisotropy values in the hippocampal regions may be the result of chronic stress-induced myelin damage and compensation from nearby brain regions acting together. After XYS intervention, fractional anisotropy values in the hippocampal regions showed a trend closer to normal levels, further validating that XYS intervention can alleviate microstructural damage in the hippocampus caused by depression.

## Conclusions

The current study highlights that the antidepressant efficacy of XYS may be intricately linked to its ability to regulate gut microbiota and microbial metabolites, thereby influencing the spatial distribution and concentration of brain tissue-specific functional metabolites. This modulation contributes to metabolic reprogramming of the gut-brain axis. Furthermore, the study elucidates tissue-specific changes in metabolic pathways—including lipid metabolism, neurotransmitter metabolism, and energy metabolism—associated with the pathogenesis of depression. It also explores the interplay between gut microbes, metabolite alterations, and depression-like behaviors, providing enriched insights into the molecular basis of depression. These abnormal metabolic brain regions, particularly the prefrontal cortex and hippocampus regions, showed significant changes in their corresponding diffusion indicators, suggesting that the microscopic structural properties (integrity of myeline and synapses) were disrupted. XYS can alleviate the degree of disruption in these regions. These findings enhance our understanding of the complex pathophysiology of depression and establish a molecular framework for advancing the development of the herbal formula XYS as a potential therapeutic intervention for depression.

## Supplementary Information

Supplementary material 1.

## Data Availability

No datasets were generated or analysed during the current study.

## Abbreviations

5-HT: Serotonin
ACH: Acetylcholine
CRS: Chronic restraint stress
DTI: Diffusion tensor image
EPM: Elevated plus-maze test
FLX: Fluoxetine
GABA: γ-Aminobutyric acid
HE: Hematoxylin and eosin
LysoPC: Lysophosphatidylcholine
mPFC: Medial prefrontal cortex
MRI: Magnetic resonance imaging
OPLS-DA: Orthogonal partial least squares-discriminant analysis
OFT: Open field test
PA: Phosphoric acid
PC: Phosphatidylcholine
PE: Phosphatidylethanolamine
SPT: Sucrose preference test
SM: Sphingomyelin
TCM: Traditional Chinese medicine
TST: Tail suspension test
XYS: Xiaoyaosan
