# Supplementary material for: Xiaoyaosan modulates gut-brain metabolic pathways and brain microstructure in depression: a multi-omics insight
Source: Chin Med. 2025 Oct 1;20:151. doi: 10.1186/s13020-025-01212-z (PMC12486543; doi:10.1186/s13020-025-01212-z)
Supplement: Supplementary file 1 — Supplementary material 1. [file 13020_2025_1212_MOESM1_ESM.docx]

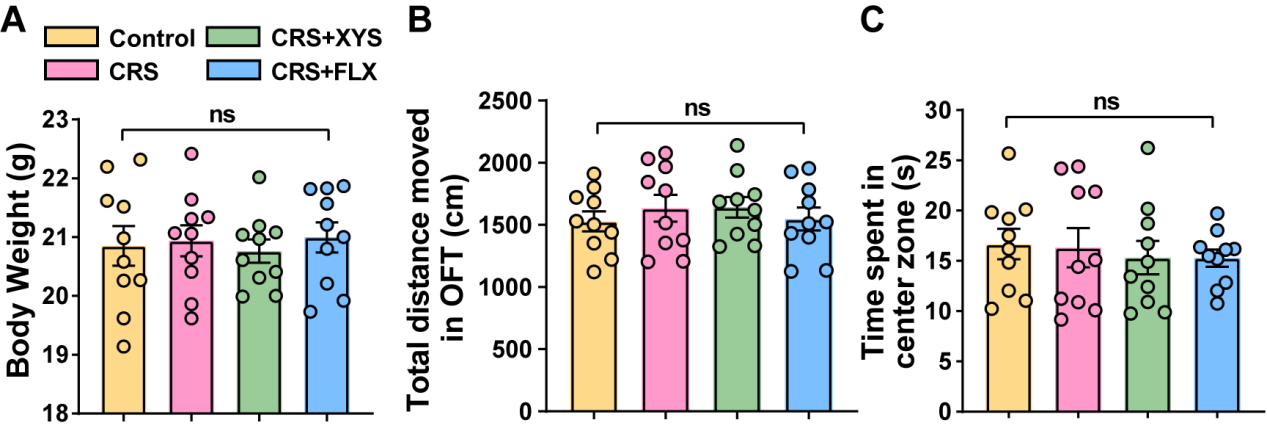


**Figure S1：**Prior to the CRS experiments, no significant difference was observed among all groups. (A)The body weight. (B)The total distance moved in OFT. (C) The time spent in center zone in OFT.


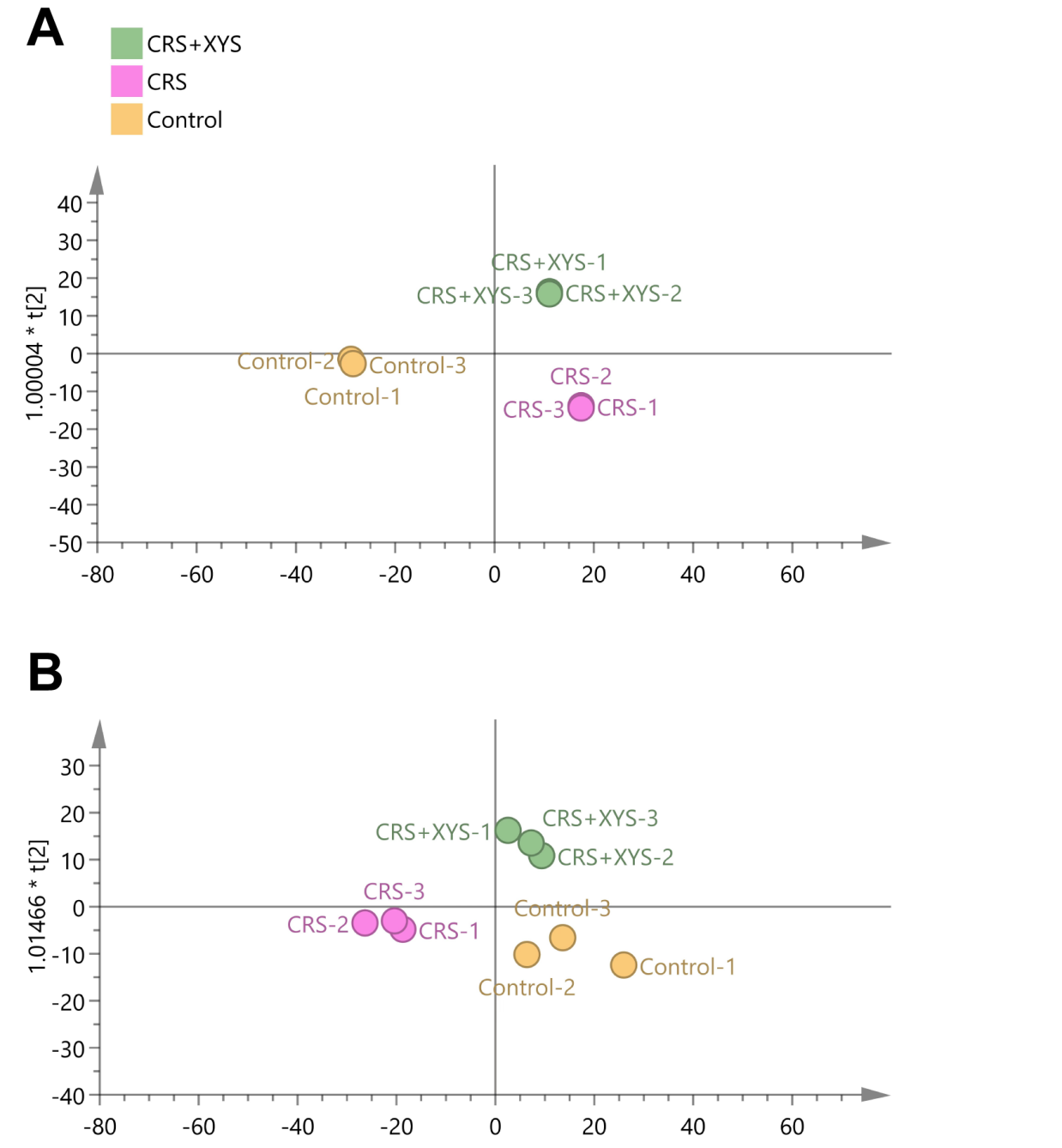


**Figure S2：**OPLS-DA score scatter plots for the pairwise comparisons between the control and CRS groups. (A) Positive mode. (B)Negative mode.
